# Ushering in a new era of single-cell transcriptomics in bacteria

**DOI:** 10.1093/femsml/uqac020

**Published:** 2022-09-21

**Authors:** Christina Homberger, Lars Barquist, Jörg Vogel

**Affiliations:** Institute of Molecular Infection Biology, University of Würzburg, D-97080 Würzburg, Germany; Faculty of Medicine, University of Würzburg, D-97080 Würzburg, Germany; Helmholtz Institute for RNA-based Infection Research (HIRI), Helmholtz Center for Infection Research (HZI), D-97080 Würzburg, Germany; Institute of Molecular Infection Biology, University of Würzburg, D-97080 Würzburg, Germany; Faculty of Medicine, University of Würzburg, D-97080 Würzburg, Germany; Helmholtz Institute for RNA-based Infection Research (HIRI), Helmholtz Center for Infection Research (HZI), D-97080 Würzburg, Germany

**Keywords:** single-cell RNA-seq, heterogeneity, microSPLiT, PETRI-seq, MATQ-seq, par-seqFISH

## Abstract

Transcriptome analysis of individual cells by single-cell RNA-seq (scRNA-seq) has become routine for eukaryotic tissues, even being applied to whole multicellular organisms. In contrast, developing methods to read the transcriptome of single bacterial cells has proven more challenging, despite a general perception of bacteria as much simpler than eukaryotes. Bacterial cells are harder to lyse, their RNA content is about two orders of magnitude lower than that of eukaryotic cells, and bacterial mRNAs are less stable than their eukaryotic counterparts. Most importantly, bacterial transcripts lack functional poly(A) tails, precluding simple adaptation of popular standard eukaryotic scRNA-seq protocols that come with the double advantage of specific mRNA amplification and concomitant depletion of rRNA. However, thanks to very recent breakthroughs in methodology, bacterial scRNA-seq is now feasible. This short review will discuss recently published bacterial scRNA-seq approaches (MATQ-seq, microSPLiT, and PETRI-seq) and a spatial transcriptomics approach based on multiplexed *in situ* hybridization (par-seqFISH). Together, these novel approaches will not only enable a new understanding of cell-to-cell variation in bacterial gene expression, they also promise a new microbiology by enabling high-resolution profiling of gene activity in complex microbial consortia such as the microbiome or pathogens as they invade, replicate, and persist in host tissue.

## Main text

Single-cell RNA-sequencing (scRNA-seq) is revolutionizing biology and medicine, combining the advantages of bulk sequencing techniques and microscopic analysis of individual cells (Jovic et al. 2022). It has catalyzed the discovery of new cell types and provided an unprecedented view of tissue anatomy and cellular transitions (Stubbington et al. 2017). While eukaryotic scRNA-seq protocols have generally taken advantage of the same next-generation sequencing (NGS) technologies that have enabled fast and cost-effective whole genome sequencing, they have evolved quickly thanks to the introduction of additional specialized platforms and protocols, e.g. the droplet-based sequencing 10x Genomics platform (Zheng et al. 2017), Smart-Seq (Ramsköld et al. 2012, Picelli et al. 2013, Hagemann-Jensen et al. 2020), or BD microwell-array-based sequencing (Fan et al. 2015, Mair et al. 2020). These workflows usually target polyadenylated RNAs and are, therefore, tailored to scRNA-seq of eukaryotic cells.

NGS technologies including RNA-seq have also opened a new window on the diversity and complexity of microbes (Westermann and Vogel 2021), and thereby contributed to the current renaissance of molecular microbiology. While the classical view of bacterial gene expression was relatively simple, the emerging view is more complex, encompassing extensive post-transcriptional control and regulatory networks (Wade and Grainger 2014, Georg and Hess 2018, Hör et al. 2018, Holmqvist et al. 2018, Adams and Storz 2020, Ponath et al. 2022). Moreover, increasing interest in the human microbiota and environmental microbial communities has highlighted the importance of understudied bacterial species with unknown transcriptome structures. While the bulk RNA-seq methodologies underlying these discoveries have revolutionized the field, understanding how regulatory networks and bacterial communities are organized at the level of single cells remains technically challenging.

Single-cell transcriptomics has the potential to move beyond bulk methods and provide direct insight into the differences in expression between individual cells within a community (Brennan and Rosenthal 2021, Lloréns-Rico et al. 2022). Cell heterogeneity allows clonal communities to develop a complex array of phenotypes (Elowitz et al. 2002, Ackermann 2015), such as production of persisters that resist antibiotic treatment, the formation of spatially organized biofilms, or the development of metabolically specialized cells under nutrient-limiting conditions (Stewart and Franklin 2008, Gollan et al. 2019). Fluorescent reporter constructs have helped to visualize heterogeneous gene expression within bacterial populations (Roche and Bumann 2021). However, such reporters are limited to assaying single genes in a few genetically tractable model species, while the vast majority of bacteria remain difficult to engineer and or even cultivate.

While bulk RNA-seq approaches capture the average gene expression signal across large cell populations, they cannot provide information on heterogeneity in transcriptional output between individual bacteria. Similarly, initial attempts to establish global gene expression profiling for single (Kang et al. 2011, 2015, Wang et al. 2015) or small numbers of bacterial cells (Avital et al. 2017, Penaranda and Hung 2019) struggled to infer the physiological state of individual bacteria within a population. Bacterial scRNA-seq opens a large collection of research topics to study (Box 1). As will be described in this short review, global transcriptomics in single bacteria has now been firmly established, but has had to overcome several major hurdles, briefly introduced in the following.

Box 1: **Examples of research topics to benefit from bacterial single-cell transcriptomics.**

The promises of bacterial scRNA-seq for microbiology and infection biology are manifold. Phenotypic heterogeneity of genetically identical bacteria has been observed in numerous studies in the area of microbiology and infection biology (Striednig and Hilbi 2022). For example, biofilms formed by uropathogenic *E. coli* exhibit distinct subpopulations showing heterogeneous expression of respiratory complexes (Beebout et al. 2019) and differentiation into various phenotypic cell states (Yannarell et al. 2021). Quorum sensing, a molecular process that regulates biofilm formation, shows heterogeneity especially in the early phase of biofilm lifecycle (Cárcamo-Oyarce et al. 2015). Phenotypic heterogeneity has also been reported in reference to antibiotic tolerance and stress responses. Examples include heterogeneous expression of flagellar genes and antibiotic tolerance under Ciprofloxacin exposure in *S. enterica* (Lyu et al. 2021); and heterogeneity in stress responses such as the SOS response in *E. coli* and *S. enterica* (Jones and Uphoff 2021, Mérida-Floriano et al. 2021, Jaramillo-Riveri et al. 2022, Sampaio et al. 2022). Bacterial secretion systems also show highly heterogeneous expression patterns within genetically identical populations. One prominent example is the type-III secretion system (T3SS) found in many Gram-negative bacteria. Animal experiments, where mice infected with *S. enterica* revealed an ON/OFF state of the energetically costly T3SS expression, even though it is required for infection, indicative of a ‘self-destructive cooperation’ (Ackermann et al. 2008). Such heterogeneity seems to be a common feature of host–pathogen interactions (Bumann 2015). A particularly important example of this is in persister formation. Persisters are a small subpopulation of bacteria that can withstand antibiotic exposure and cause infection relapse (Fisher et al. 2017). The underlying mechanisms leading to this state remain unclear. However, within their host niche, persister cells seem far from transcriptionally silent (Stapels et al. 2018). Bacterial scRNA-seq of pathogenic persisters isolated from infected patients could help us understand their *in vivo* activities and reveal when, how, and why they reactivate their gene expression and metabolic state.

### Technical challenges for scRNA-seq in bacteria

**Figure 1.**
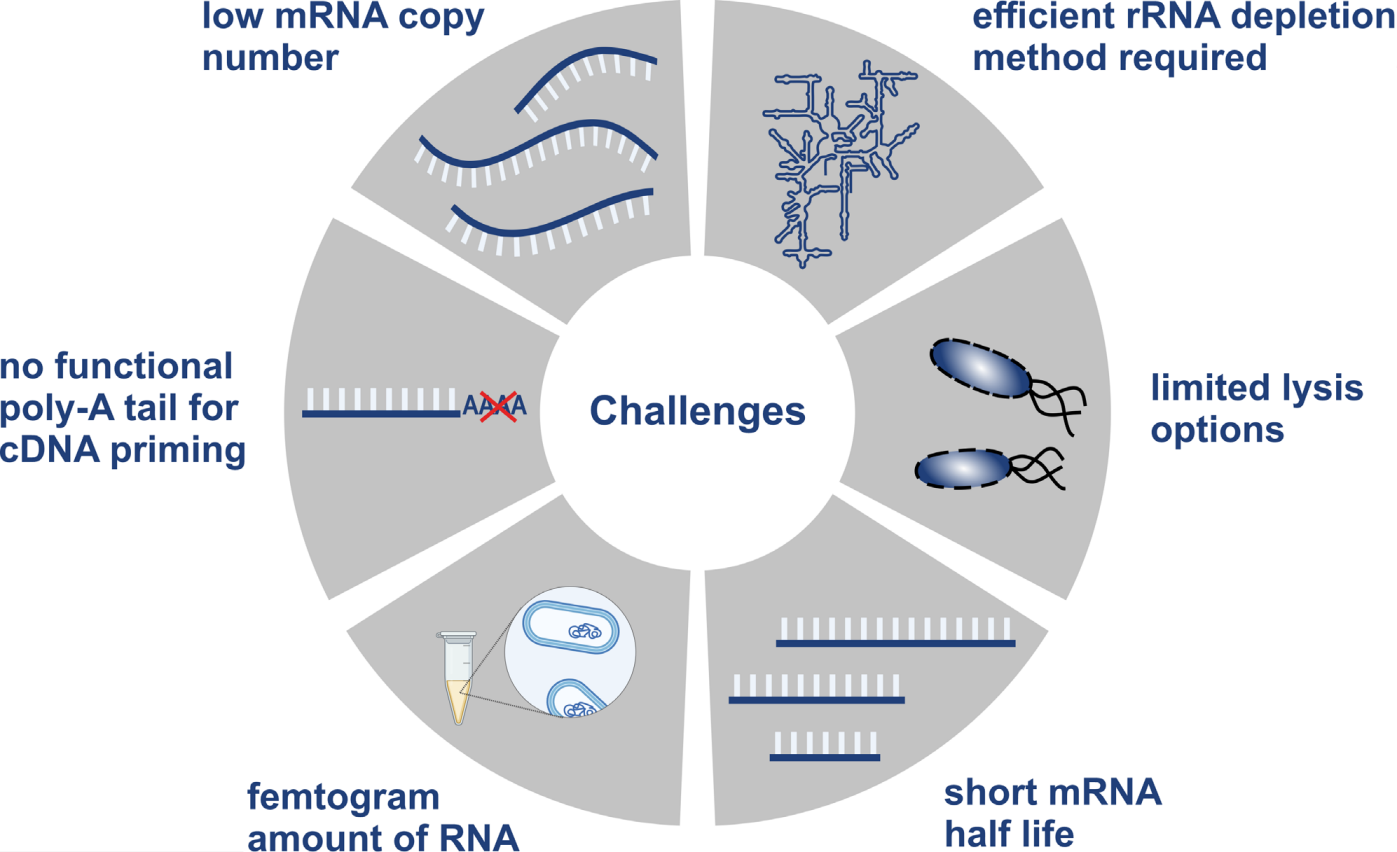
Challenges in bacterial single-cell transcriptomics. Bacterial single-cell transcriptomic studies come along with various challenges related to the specific features of bacterial mRNA, cell wall composition, and cell scale. Thus, method development requires consideration of technical methods for resolution of these challenges. Created with Biorender.com.

In establishing single-cell transcriptomics workflows in bacteria, several technical challenges had to be considered in the selection of suitable protocols (Fig. 1). Because bacterial cells contain only femtogram amounts of RNA (Milo and Phillips 2015), which is > 100-times less than a typical eukaryotic cell, a very sensitive cDNA synthesis and amplification protocol is required. In addition, bacterial mRNAs are intrinsically labile, displaying half-lives in the minute range, as compared to hours in eukaryotes. This necessitates rapid perforation of the thick bacterial envelope, cell lysis, and subsequent RNA stabilization. At the same time, the cell lysis procedure has to be highly efficient in order to extract mRNAs from single bacterial cells; it is fair to assume that lysis efficiency anticorrelates with the RNA retention rate, i.e. the amount of RNA available per cell after lysis to serve as template for reverse transcription (RT). A low retention rate due to differences in lysis efficiency risks biasing resulting data against certain cell states, or the loss of certain species during library preparation from multibacteria communities. It is important to bear in mind that some of the typical lysis procedures for bulk RNA-seq, such as the use of guanidinium thiocyanate (as in the popular TRIzol reagent and variants thereof), bead beating, sonication, or column-based extraction are largely incompatible with the workflow of bacterial scRNA-seq. For example, guanidinium thiocyanate would inhibit the downstream cDNA synthesis steps when scRNA-seq is performed as a single-tube reaction. Finally, washing and rebuffering steps must be kept to a minimum so as not to risk loss of the minute amount of RNA that can be extracted from a single bacterium.

Another important step in RNA-seq workflows is the removal of the most abundant class of RNA, ribosomal RNA (rRNA), which constitutes up to 98% of a bacterium’s total RNA (Giannoukos et al. 2012, Westermann et al. 2016) and is generally not informative of cellular state. However, the absence of a poly(A) tail on bacterial transcripts precludes a straight-forward RT using poly-T primers to selectively enrich mRNAs and concomitantly deplete rRNA, as generally done for eukaryotic scRNA-seq. Therefore, the majority of available protocols for eukaryotic scRNA-seq cannot be used for bacteria without major modifications. The average transcript concentration in bacterial cells is also an important consideration: whereas most current eukaryotic scRNA-seq protocols have a lower transcript detection limit of 10 copies per transcript per cell (Ziegenhain et al. 2017, Bagnoli et al. 2018), bacterial scRNA-seq must take into account the much lower average mRNA copy-number in these organisms [0.4 copies/cell according to Taniguchi et al. (2010), Bartholomäus et al. (2016)]. Thus single-cell transcriptomics in bacteria requires a highly sensitive RNA-seq protocol; ideally, this protocol would be generic in the sense that it can be applied to diverse bacterial species and so used for the analysis of mixed microbial communities.

### New protocols for bacterial single-cell transcriptomics

**Figure 2.**
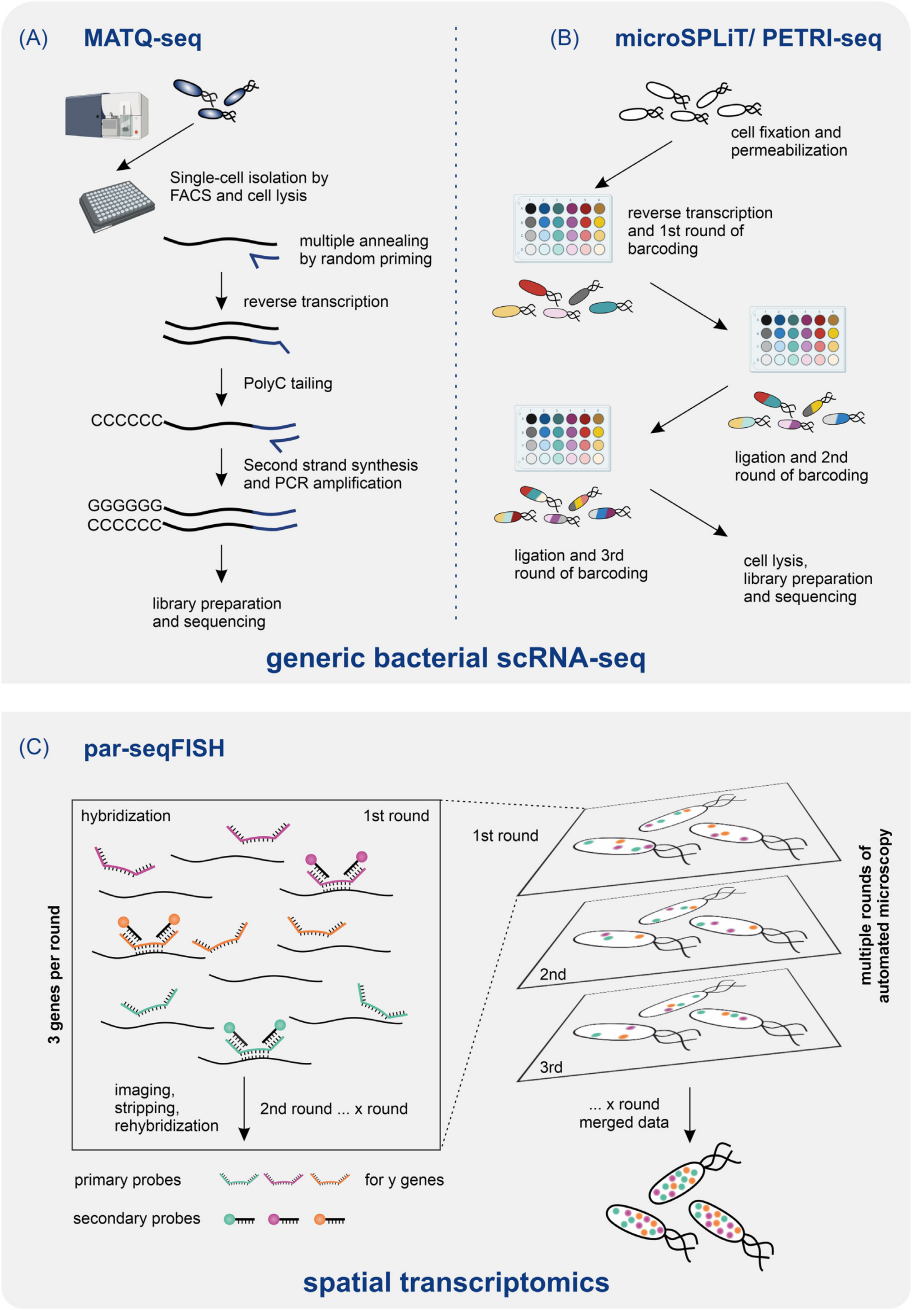
Bacterial scRNA-seq workflows. Generic bacterial scRNA-seq workflows: (A) MATQ-seq is a multiple annealing and tailing-based workflow, which enables targeting of low abundant transcripts in single bacteria. After RT, poly-C tailing, second strand synthesis, and PCR amplification, cDNA suitable for library preparation and sequencing is generated. (B) microSPLiT as well as PETRI-seq are based on split-pool barcoding system. After permeabilization and fixation of cells, three rounds of barcoding using RT and ligation process are performed. Thereby, each cDNA within a cell receives a unique barcode combination, which is used as cell identity. Barcoding is followed by cell lysis, library preparation, and sequencing. (C) Spatial transcriptomics of single bacteria: par-seqFISH is based on sequential FISH technology using sets of primary and secondary probes specifically targeting a selection of genes for high-throughput microscopy. Rounds of hybridization and stripping of three probes per cycle allow detection of gene expression profiles gene by gene. An overlay of the generated images results in a merged transcriptome map providing gene expression profiles of individual bacteria including spatial information.

Over the past 2 years, four different protocols for bacterial single-cell transcriptomics have been published (Fig. 2). As discussed below, these protocols have proven the feasibility of scRNA-seq for both Gram-negative and -postive bacteria, including both major human pathogens and model bacteria (Blattman et al. 2020, Imdahl et al. 2020, Dar et al. 2021, Kuchina et al. 2021). One of these studies adapted eukaryotic Multiple Annealing and dC-Tailing-based Quantitative scRNA-seq (MATQ-seq; Sheng et al. 2017) to profile individual *Salmonella enterica* and *Pseudomonas aeruginosa* cells after cell sorting (Imdahl et al. 2020). Benchmarking with established bulk RNA-seq data for *S. enterica* revealed that these single-bacterium transcriptomes faithfully capture condition-dependent gene expression patterns. A total of two other studies introduced protocols based on permeabilization of cell membranes followed by repeated pooling and *in situ* ligation (so-called microbial Split-Pool Ligation Transcriptomics (microSPLiT) and Prokaryotic Expression profiling by Tagging RNA *In situ* and sequencing (PETRI-seq) to study individual Gram-negative (*Escherichia coli*) and Gram-positive (*Bacillus subtilis*, *Staphylococcus aureus*) bacteria (Blattman et al. 2020, Kuchina et al. 2021)). Both approaches build on a protocol previously developed for eukaryotic scRNA-seq that uses combinatorial indexing to barcode transcripts *in situ*, a method originally known as ‘SPLiT-seq’ (Rosenberg et al. 2018). Besides bacterial scRNA-seq, gene expression analysis can also be combined with spatial context information using Parallel Sequential Fluorescence *In situ*Hybridization (par-seqFISH). The par-seqFISH technique allowed for the transcriptome imaging of more than 100 marker genes in *P. aeruginosa* and identified distinct phenotypic structuring and variation during different stages of biofilm formation (Dar et al. 2021). The following sections will cover these four protocols in more technical detail.

### MATQ-seq: multiple annealing and tailing-based microbial scRNA-seq

In the MATQ-seq protocol, total RNA recovered after single-cell lysis undergoes RT using a combination of random-hexamer and Multiple Annealing and Looping-based Amplification Cycles (MALBAC) primers. MALBAC primers, originally used for whole-genome amplification, enable high efficiency in hybridization with transcripts even at low temperatures, thereby targeting even low abundant transcripts. Those serve as primers for quasilinear preamplification of RT during MATQ-seq, which leads to a reduction of amplification bias by excluding full amplicons as templates for subsequent polymerase chain reaction (PCR) cycles (Zong et al. 2012). The RT reaction is followed by dC-tailing facilitating efficient second-strand synthesis. Finally, the cDNA is amplified during several rounds of PCR and serves as input for Nextera XT (Illumina) library preparation. Compared to the originally published MATQ-seq protocol implemented for eukaryotic cells (Sheng et al. 2017, Sheng and Zong 2019), major adaptations were required for bacteria in both the cell isolation and lysis strategy as well as the data analysis pipeline (Imdahl et al. 2020).

In the published bacterial MATQ-seq protocol (Imdahl et al. 2020), the isolation of bacterial cells is performed using fluorescent activated cell sorting (FACS) and cells are sorted directly into multiwell plates granting separation of each cell before lysis. Bacterial MATQ-seq captures around 150–200 different mRNAs per single bacterium. On the face of it, this is only ∼5% of all mRNAs in a typical bacterium (Bartholomäus et al. 2016). However, it is two orders of magnitude more than what was achieved with fluorescent gene reporters and these numbers sufficed to identify specific transcriptional signatures in *S. enterica* associated with three different growth and stress conditions (Imdahl et al. 2020). Additionally, MATQ-seq also captures small regulatory RNAs (sRNAs), which can serve as proxies for the activity of certain stress responses or virulence programmes (Papenfort and Vogel 2014, Fröhlich and Gottesman 2018, Hör et al. 2020). MATQ-seq has been successfully applied to two bacterial species, *S. enterica* and *P. aeruginosa* (Imdahl et al. 2020), which are sufficiently different to suggest that this scRNA-seq protocol has the potential for application to many other bacteria.

### PETRI-seq and microSPLiT: barcoding based scRNA-seq of microbes

Both microSPLiT (Kuchina et al. 2021) and PETRI-seq (Blattman et al. 2020) build upon the previously developed eukaryotic SPLiT-seq method (Cao et al. 2017, Rosenberg et al. 2018), so their workflows are very similar. Bacteria are immobilized by fixation and subsequently permeabilized with the help of either lysozyme or lysostaphin (PETRI-seq) or a combination of lysozyme and Tween-20 (microSPLiT). The permeabilization allows for the introduction of barcoded random hexamers, RT enzymes and ligases for subsequent *in situ* split-pool barcoding. The combinatorial barcoding requires three major experimental steps: (1) splitting of the bacteria into 96-well plates containing well-specific barcodes; (2) introduction of a well-specific barcode through RT (in the first round) or ligation (in the second and third rounds); and (3) cell pooling before splitting for next round of barcoding. This repeated splitting, tagging, and pooling procedure leads to the RT products within each individual cell being labelled with a unique combination of barcodes that serves as a cell identification. After pooling at the end of the third round of barcoding, cells are lysed to access the cDNA, i.e. then used as input for library preparation. Library preparation for microSPLiT is performed with NGS Library Construction Reagents from Enzymatics, while PETRI-seq uses the Nextera XT kit distributed by Illumina. In microSPLiT, there is an additional enrichment step for mRNAs using the polyadenylate polymerase (PAP) enzyme, which preferentially polyadenylates mRNAs. In contrast to MATQ-seq, these two barcoding-based scRNA-seq approaches do not require physical separation of cells using microfluidics or cell sorters. Instead, the cell itself serves as a reaction compartment where RT, barcoding, and cDNA synthesis are performed.

Both split and pool-based studies profiled one Gram-negative (*E. coli*) and one Gram-positive (*B. subtilis* or *S. aureus*) bacterium each to prove applicability beyond a single species. Both methods can capture in the range of 100 different mRNAs per single bacterium. In the microSPLiT study, Kuchina et al. (2021) analyzed > 25 000 bacteria, successfully separating bacterial subpopulations based on their transcriptional profiles and in *B. subtilis* identifying rare cell states induced by cellular stress. The PETRI-seq study (Blattman et al. 2020) focused on the analysis of different growth-conditions and revealed growth-dependent subpopulations via their distinct single-cell transcription profiles in *E. coli*. This study also identified rare cell states related to phage lysis.

### par-seqFISH: spatial transcriptomics in bacterial communities

The development of spatial transcriptomics has a history spanning several decades since the invention of *in situ* hybridization, initially to detect rRNA (Moses and Pachter 2022). Despite this, conventional fluorescence-*in situ* hybridization (FISH)-based technologies were long restricted to measuring the expression of only a few genes at a time (Fei and Sharma 2018). However, the recently developed sequential FISH (seqFISH) technique (Eng et al. 2019) increased this number to thousands of genes, at least in eukaryotes, by combining FISH with combinatorial imaging. For the fluorescent-based readout of seqFISH, a set of primary probes are designed complementary to a selection of target genes. The probes include unique flanking regions, which enable gene-specific binding of a single secondary, or readout, probe coupled to a fluorophore. By using different fluorophores and performing several rounds of hybridization, imaging, and stripping of the secondary probes, the expression of many different genes can be detected consecutively in a single sample.

In developing par-seqFISH, the original eukaryotic seqFISH protocol was refined and successfully adapted for complex bacterial communities (Dar et al. 2021). As to major challenges to overcome, the small size of bacteria did not allow the standard ‘barcoding’ approach, wherein several probes are applied to read the expression of multiple genes simultaneously. Instead, par-seqFISH uses a parallelization step where three orthogonal fluorophores are used at once to read out three genes per hybridization round. Thereby par-seqFISH was shown to capture transcripts across a wide range of expression levels without interference from the abundant rRNA. In addition to this parallelization and cell-level barcoding procedure for multiplexing, par-seqFISH uses automated microscopy to generate a sequence of images capturing the gene expression through sequential application of different secondary probe sets, which further helps to achieve high throughput. To correct for underestimation of the transcript count due to the merging of fluorescent signal from spatially close transcript copies, continuous intensity values were digitized using the fluorescent signal from low-expression genes. This digitization provides an estimate of the transcript copy number at each fluorescent spot. Overlaying the images from many rounds of secondary hybridization resulted in a merged data set that provides transcriptomic measurements on a spatial scale with single-cell resolution.

The pioneering par-seqFISH study (Dar et al. 2021) investigated *P. aeruginosa*, an opportunistic pathogenic bacterium associated with nosocomial infections and a model organism for studying biofilm formation. Primary and secondary probes were designed for a selection of 105 marker genes to study various planktonic populations as well as heterogeneity in different biofilm states. In total, the transcriptional profiles of > 600 000 *P. aeruginosa* cells were analyzed. This data revealed distinct metabolic and virulence-related subpopulations in planktonic growth conditions. Comparing biofilm with planktonic lifestyle, the authors identified several transcriptional responses specific to biofilms. Specifically, they found that sessile cells can be defined by the expression of matrix component genes not observed in planktonic cells. Moreover, they found differences in spatial architecture distinguishing early and mature biofilms, defined by distinct expression patterns of proteases and quorum sensing regulatory networks (Dar et al. 2021).

### Comparison and development of different methods

The four methods discussed above come with their own strengths and weaknesses, as summarized in Table 1. MATQ-seq has a much lower drop-out rate (percentage of cells lost during sample processing and/or excluded by bioinformatic filtering) than have PETRI-seq and microSPLiT, while the combinatorial barcoding-based methods offer a much higher throughput. MATQ-seq showed a consistently high number of genes detected per single bacterium and a very high lysis efficiency due to optimization for each species. In contrast, PETRI-seq and microSPLiT had a lower transcript recovery rate but used a more generic lysis protocol with broader applicability. However the different filtering and transcript detection cut-off criteria applied in each data analysis pipeline, as well as the different bacterial species investigated in each study, makes a direct comparison challenging at this point in time. The strength of par-seqFISH is its ability to reveal spatial dynamics by preserving the physical context of bacteria. However, par-seqFISH is limited by the number of probes, and each probe set added requires an additional washing and hybridization step. This may have implications for detecting subpopulations of cells expressing genes not previously associated with a condition of interest by bulk RNA-seq.

**Table 1:** Characteristics of approaches to bacterial single-cell transcriptomics.

Categories	MATQ-seq	microSPLiT	PETRI-seq	par-seqFISH
Probe-independent approach	Yes	Yes	Yes	No
Cell throughput	Hundreds of cells	Thousands of cells	Thousands of cells	Thousands of cells
Sensitivity^#^	150–200 genes (*S. enterica*)	230 genes (*B. subtilis*); 138 genes (*E. coli*)	103 operons (*E. coli*)	105 genes (probe-dependent)
Cell drop-out rate	Low	High	High	Low
Costs	High	Moderate	Moderate	Moderate
Applied organisms	*S. enterica*,*P. aeruginosa*	*E. coli*,*B. subtilis*	*E. coli*,*S. aureus*	*P. aeruginosa*

# Number of detected genes is highly dependent on applied cut-off criteria.

Regarding the high prevalence of noninformative rRNA, only microSPLiT includes a dedicated enrichment step for mRNA. There, a separate polyadenylation using PAP is performed after permeabilization. However, a targeted rRNA depletion protocol is currently missing for bacterial scRNA-seq. We have recently achieved efficient depletion of rRNA reads from bacterial cDNA using the Cas9 nuclease (Prezza et al. 2020) and successfully implemented it in the MATQ-seq workflow (own unpublished results). Generally speaking, such rRNA depletion step will substantially lower the cost of bacterial scRNA-seq experiments and while also increasing the proportion of mRNA reads at the same time, thus improving mRNA detection rates. Future improvements of the current protocols should also focus on the optimization of turn-around times and the implementation of automation steps in sample preparation as well as liquid handling. A more broadly applicable lysis protocol, higher cell throughput (for MATQ-seq) and a lower drop-out rate (for barcoding based workflows) would be desirable. For par-seqFISH we are yet to see how this method will perform with an organism other than *P. aeruginosa*.

## Conclusion and outlook

The proof-of-concept studies discussed here have firmly established that transcriptomes can be read from individual bacterial cells. These new methods promise a new single-cell microbiology that seeks to fathom cellular heterogeneity in microbial populations and its consequences for adaptation to diverse conditions including infection and other multiorganism interactions. Fulfilling this promise will require further developments to increase throughput, capture temporal dynamics, and allow for direct probing of multicellular interactions.

The development of microfluidic methods for cell isolation and library preparation was one of the major factors driving the exponential scaling in the number of cells analyzed by eukaryotic scRNA-seq over the past decade (Svensson et al. 2018). In particular, the development of reliable microfluidic droplet sequencing, in which cells are individually encapsulated in emulsion droplets that serve as vessels for RT, has made scRNA-seq experiments measuring gene expression in thousands of cells simultaneously increasingly routine (Klein et al. 2015, Macosko et al. 2015, Zheng et al. 2017). However, these methods are still in their infancy for bacteria, mainly due to a lack of both universal droplet-compatible bacterial cell lysis techniques and robust RT protocols that do not depend on transcript polyadenylation. Of note, two recent preprints describe the successful use of the commercial 10x Genomics platform for bacterial scRNA-seq (McNulty et al. 2021, Ma et al. 2022). In the future, successful development of universal droplet-based methods may lead to a general pipeline for high-throughput bacterial scRNA-seq that retains the sensitivity of MATQ-seq, while approaching the throughput enabled by split and pool protocols. Very recently, single-microbe genomics at the level of DNA using single-cell droplet encapsulation has been published and lends itself for adaptation to bacterial scRNA-seq (Zheng et al. 2022).

One major limitation of most ‘omics approaches, scRNA-seq included, is that the profiles generated only capture a single point in time, obscuring the underlying dynamics of the system under study. As we are generally interested in dynamic processes, for instance metabolism within a single cell or during host cell invasion, it would be helpful if we could extract some indication of how the system evolves over time. In the study of eukaryotic cells, the concept of RNA velocity has been used to achieve this with wide application (Bergen et al. 2021). Conceptually, RNA velocity uses the ratio of spliced to newly transcribed unspliced mRNA to provide an estimate of the instantaneous change in transcription at the time the sample was taken. As bacterial mRNA is generally not spliced during maturation, such techniques would not be directly transferrable. However, there are existing protocols that profile nascent transcripts through whole-cell metabolic labelling (Erhard et al. 2022), which allows for the marking of newly synthesized RNA through incorporation of artificial modified nucleotides. One of these methods is Thiol(SH)-linked Alkylation for the Metabolic Sequencing of RNA (SLAM-seq; Herzog et al. 2017) that uses metabolic labelling using 4-thiouridine, which can be converted to cytidine by chemical treatment, allowing differentiation between newly transcribed and old RNA by the presence of T to C conversions in sequencing reads. Extending this concept to single cells, scSLAM-seq (Erhard et al. 2019) enabled the analysis of rapid cellular responses of individual cells to viral infections. New methods for metabolic labelling of RNA in bacteria suggest a similar approach could be extended to bacteria (Meng et al. 2020). A modified scSLAM-seq protocol for bacteria would be a promising tool to understanding the transcriptional adaptation that occurs upon entrance to a new environment, for instance a host cell. However, it is important to consider the short half-lives of bacterial mRNAs (in the minute range) as limiting time factor for capturing temporal transcriptional changes.

Host–microbe interactions involve multiple interaction partners, and scRNA-seq has already revealed the existence of complex signalling pathways and a high level of transcriptional heterogeneity in infected host cells (Avraham et al. 2015, Saliba et al. 2016). Simultaneous bulk transcriptome profiling of pathogen and host has become a popular approach to investigating the interactions that occur during infection (Tsai and Coombes 2019, Westermann and Vogel 2021). These multiorganism RNA-seq approaches (Dual RNA-seq, Triple RNA-seq) are based on poly(A)-independent library preparation protocols and capture all transcript classes (Westermann et al. 2016, Westermann and Vogel 2018, Seelbinder et al. 2020), but lack the sensitivity necessary to be performed on the level of single cells. A number of approaches have been taken towards establishing Dual RNA-seq with single-cell resolution. For instance, the single-cell Dual RNA-seq (scDual-Seq) method isolates single infected eukaryotic cells by FACS and uses random hexamers to prime both host and pathogen-derived transcripts. Subsequent poly-A tailing and *in vitro* transcription allows simultaneous transcriptome analysis of host and pathogen (Avital et al. 2017). In contrast, the Pathogen Hybrid-Capture (PatH-Cap) method specifically enriches bacterial mRNA over host RNA through capture with targeted oligonucleotides. PatH-Cap is sensitive enough to capture the transcriptome of a few bacterial cells within a single host cell (Betin et al. 2019). However, neither method provides insight into potential heterogenous gene expression among multiple intracellular bacteria within the same host cell. For this, the development of new methods and refinement of existing methods is required. One of the major challenges will be to capture efficiently RNA of both eukaryotic and prokaryotic cells with unbiased efficiency and resolution. The spatial transcriptomics techniques reviewed here may provide an intermediate step to whole transcriptome capture of individual bacteria from intracellular communities.

It seems unlikely that any single technique will come to dominate the bacterial scRNA-seq field in the immediate future. As in eukaryotes, where multiomics approaches that layer information from suites of assays are becoming increasingly common (Sharma et al. 2020, Li et al. 2021, Conrad and Altmüller 2022), the bacteriology community will benefit from having access to a range of single-cell technologies with different strengths and weaknesses. For instance, with the technologies discussed in this review, one could already imagine applying split and pool methods to identify highly expressed marker genes in a large number of cells within a complex population, then using these markers to map the spatial organization of cells using par-seqFISH, before using MATQ-seq to produce high resolution transcriptomes for selected cells with characteristics of interest. As these technologies mature and new ones are added, methods for data integration will become increasingly important, as can be seen in the current transformation of the eukaryotic single-cell field from being driven primarily by experimental techniques to one increasingly reliant on advanced data science. While many technical challenges remain to be solved to create production-ready bacterial scRNA-seq protocols that can be applied on the industrial scales currently being undertaken for eukaryotic scRNA-seq in projects like the Human Cell Atlas (Rozenblatt-Rosen et al. 2017), the studies reviewed here have already made significant progress towards opening the microbial world to investigation on the scale of the fundamental unit of life: the single cell.
